# Evaluation of the efficacy of intrathecal injection of amitriptyline and doxepin in spinal anesthesia in comparison with bupivacaine in rats

**DOI:** 10.5812/kowsar.22287523.1511

**Published:** 2011-07-01

**Authors:** Mahmoud Reza Alebouyeh, Farnad Imani, Poupak Rahimzadeh, Seyyed Hamid Reza Faiz

**Affiliations:** 1 Department of Anesthesiology and Pain Medicine, Rasoul-Akram Medical Center, Tehran University of Medical Sciences, Tehran, IR Iran; 2 Department of Anesthesiology, Rasoul-Akram Medical Center, Tehran University of Medical Sciences, Tehran, IR Iran

**Keywords:** Doxepin, Amitriptyline, Bupivacaine, Spinal anesthesia

## Abstract

**Background:**

Tricyclic antidepressants (TCAs) are commonly used orally for treating chronic pain states, such as neuropathic pain. TCAs produce analgesia by various mechanisms, including sodium channels, N-methyl-d-aspartate receptors, biogenic amines, opioids, inflammatory mediators, and substance P. Studies have shown that intrathecal tricyclic administration effectively attenuates pain and thermal hyperalgesia in inflammatory and neuropathic pain in rats.

**Objectives:**

The aim of this study was to evaluate the effect of two tertiary TCAs in sensory and motor block. We also used bupivacaine as a strong local anesthetic for the control group.

**Materials and Methods:**

In a double-blind randomized controlled trial in an animal lab, intrathecal injection of drugs was performed in 30 Wistar male rats. We divided the subjects into 3 groups: group 1: 90 µL Doxepine (50 mM), group 2: 90 µl amitriptyline (60 mM). and group 3: 90 µL bupivacaine (23 mM). Then sensory, motor, and proprioceptive changes were measured at 1, 2, 3, 4, 6, and 12 hours by one examiner.

**Results:**

In Groups 1 and 2, a total of 3 rats died. After adjusting the concentrations, amitriptyline had a similar potency but a longer duration of spinal blockade of motor, proprioception, and nociception than did bupivacaine (p < 0.05), whereas doxepin had a reasonable but lower efficacy and shorter duration of spinal blockade than did bupivacaine (p < 0.05). The full recovery time for Group 2 was significantly longer.

**Conclusions:**

It seems that tertiary amine drugs such as amitriptyline and doxepin had reasonable potencies of spinal blockade when compared to bupivacaine. However, amitriptyline had a more potent and long-acting spinal anesthetic effect. Amitriptyline may turn out to be a clinically valuable local anesthetic.

## 1. Background

In modern anesthesia, neuraxial blocks have several beneficial uses. There is a tendency toward intrathecal injection of different drugs with different mechanisms by anesthetists and pain physicians. Tricyclic antidepressants (TCAs) have been found to be effective drugs in chronic pain management, especially for neuropathic pain (1). TCAs exert their effects through several complex mechanisms. One of the most important theories is analgesic induction via direct effects on NMDA receptors, biogenic amines, opioid receptors, inflammatory mediators, and substance P (2). On the other hand, inhibitory effects on sodium channels are another important mechanism for analgesia and motor block after transthecal usage (2, 3). Inhibition of norepinephrine and serotonin reuptake in terminal ending and suppressing pain transmission via ascending pathways are additional analgesic mechanisms for TCAs (3, 4). Finally, intrathecal amitriptyline and doxepin have been used in animal studies for reducing pain and heat hyperalgesia in neuropathic and inflammatory pain (3-5).

## 2. Objectives

To evaluate the theories mentioned above, we analyzed the effects of two tertiary TCAs in sensory and motor block. Bupivacaine as a strong local anesthetic was chosen for the control group. This study follows the previous literature, which mostly uses animal subjects, with an analysis on rat models.

## 3. Materials and Methods

In a double-blind, randomized clinical trial in an animal lab of Hazrat Fatemeh Medical hospital, 30 mature, male Wistar rats weighing 200-300 g were selected. Rats were healthy with no motor dysfunction. They were housed in groups of 3 for at least 1 week in a climate-controlled room maintained at 23 °C. Lighting was on a 12-h light/dark cycle with food and water available on demand except during the time of testing. All tests were performed in accordance with the recommendations, policies of the International Association for the Study of Pain and guidelines for laboratory animal experiments. Experiments were performed at the same time on light cycle in all groups. A short anesthesia was induced with intramuscular injection of 30 mg/kg ketamine and 3 mg/kg zilazine 2% (for immobility of rats). Each animal was tested only once and killed under anesthesia. After 20 minutes, with proper positioning by another colleague with needle G29 in L4-5 interspinal space, spinal approach was performed, and a Hamilton syringe injection was performed as follows:

Group 1: doxepine 90 microlitre (50 mmol);
Group 2: amitryptiline 90 microlitre (60 mmol);
Group 3: bupivacaine 90 microlitre (23 mmol).

The investigator was not aware of the syringe’s contents. Success in intrathecal injection was confirmed by a sense of ‘give’ and the sign of a tail flick. After a recovery period, a motor examination and three neurobehavioral examinations, which consisted of evaluations of motor function, proprioception, and nociception, were conducted by an expert examiner not aware of group type at 1, 2, 3, 4, 6, and 12 hours. All findings were recorded in prepared forms. Motor function was evaluated by measuring the ‘extensor postural thrust’ of the hind limbs of rats (6). To test the extensor postural thrust, the rat was held upright with the hind limbs extended so that the body weight was supported by the distal metatarsus and toes. The extensor thrust was measured as the gram-force, that resisted contacting the platform by the heel applied to a digital platform balance. The preinjection control value was measured and recorded. The reduction in force, resulting from extensor muscle tone, was considered motor deficit (Table 1). Proprioception evaluation was based on the resting posture test (6). This test was performed by lifting the front half of the animal off the ground and lifting one hind limb at a time off the ground so that the animal was standing on just one limb. Then, the animal was moved laterally with the weight-bearing limb in the direction of movement to prevent the animal from falling (Table 2). The subject’s nociceptive reaction was evaluated by the withdrawal reflex or vocalization elicited by the pinch of a skin fold over the lateral metatarsus and 5th finger distal phalanx of bilateral hind limbs (Table 2) (1, 6). All block reaction was graded as 75% and 90% maximum possible effect (MPE) (7). The results of experimented animals were evaluated by SPSS version 11 and nonparametric Mann-Witney U tests. Greenhouse-Geisser correction was used to evaluate the difference between the duration of blocks.

**Table 1.** Motor block measurement

Motor block number	Type of impairment
**0**	Normal
**1**	Mild impairment, less than 50% reduction in preinjection pressure
**2**	Severe impairment, more than 50% reduction in preinjection pressure up to 20 g
**3**	Complete block, less than 20 g pressure or paralyzed limb pressure

**Table 2.** Proprioceptive block and sensory block measurement

	Type of impairment
**Proprioceptive block number**	
**0**	Normal
**1**	Mild impairment
**2**	Severe impairment
**3**	Complete block
**Sensory block number**	
**0**	Normal, complete withdrawal and strong vocalization
**1**	Mild impairment
**2**	Severe impairment
**3**	Complete block

## 4. Results

30 rats in 3 groups were studied. One rat in first group and 2 rats in second group experienced cardiac arrest and were not included in the study. After adjusting and evaluating different concentrations of drugs, we observed that amitriptyline had the same power and ability as bupivacaine but the proprioceptive, sensory, and motor blocks were longer with this drug (Table 3). While doxepin was less effecitve for less time as a spinal anesthesia in comparison with bupivacaine, overall, the motor, proprioceptive, and sensory blocks in the amitriptyline group were significantly longer. In the amitriptyline group, 90% MPE was significantly higher (p < 0.05).

**Table 3.** Study findings in three groups (mean motor, proprioceptive, and sensory block at different hours)

	Doxepin group	Amitriptyline group	Bupivacaine group	P value
**Motor block**				
**hr 1 (Mean ± SD)**	2.2 ± 0.3	2.8 ± 0.2	2.5 ± 0.3	< 0.01
**hr 2 (Mean ± SD)**	2.1 ± 0.4	2.8 ± 0.2	2.4 ± 0.4	< 0.01
**hr 3 (Mean ± SD)**	1.1 ± 0.4	1.9 ± 0.3	1.4 ± 0.4	< 0.05
**hr 4 (Mean ± SD)**	1.1 ± 0.2	1.6 ± 0.5	1.3 ± 0.2	< 0.05
**hr 6 (Mean ± SD)**	0.1 ± 0.0	1.0 ± 0.1	0.2 ± 0.1	< 0.05
**hr 12 (Mean ± SD)**	0.0 ± 0.0	0.0 ± 0.0	0.0 ± 0.0	-
**Proprioceptive block**				
**hr 1 (Mean ± SD)**	2.3 ± 0.3	2.9 ± 0.1	2.5 ± 0.35	< 0.05
**hr 2 (Mean ± SD)**	2.3 ± 0.3	2.85 ± 0.15	2.5 ± 0.35	< 0.05
**hr 3 (Mean ± SD)**	1.5 ± 0.3	2.0 ± 0.3	1.5 ± 0.42	< 0.05
**hr 4 (Mean ± SD)**	0.4 ± 0.1	1.0 ± 0.0	0.5 ± 0.1	< 0.05
**hr 6 (Mean ± SD)**	0.0 ± 0.0	0.0 ± 0.0	0.0 ± 0.0	-
**hr 12 (Mean ± SD)**	0.0 ± 0.0	0.0 ± 0.0	0.0 ± 0.0	-
**Sensory block**				
**hr 1 (Mean ± SD)**	2.5 ± 0.3	2.9 ± 0.1	2.7 ± 0.22	< 0.05
**hr 2 (Mean ± SD)**	2.5 ± 0.3	2.9 ± 0.1	2.7 ± 0.2	< 0.05
**hr 3 (Mean ± SD)**	1.3 ± 0.1	1.85 ± 0.15	1.5 ± 0.1	< 0.05
**hr 4 (Mean ± SD)**	0.2 ± 0.1	1.0 ± 0.1	0.0 ± 0.0	< 0.05
**hr 6 (Mean ± SD)**	0.0 ± 0.0	0.4 ± 0.1	0.0 ± 0.0	< 0.05
**hr 12 (Mean ± SD)**	0.0 ± 0.0	0.0 ± 0.0	0.0 ± 0.0	-
**Motor block duration with 75% MPE (min) (Mean)**	120	150	123	< 0.05
**Proprioceptive block duration with 75% MPE (min) (Mean)**	123	170	128	< 0.05
**Sensory block duration with 75% MPE (min) (Mean)**	117	168	124	< 0.05
**With more than 90% MPE motor block (%)**	80	90	80	< 0.05
**With more than 90% MPE proprioceptive block (%)**	75	100	80	< 0.05
**With more than 90% MPE sensory block (%)**	80	100	90	< 0.05

## 5. Discussion

In comparison with bupivacaine, it seems that tertiary amines such as amitriptyline and doxepin have a reasonable ability to induce spinal anesthesia. However, previous studies have found that amitriptyline blockage is more potent and longer than bupivacaine and doxepin blockage (7, 8). The potency and duration make amitriptyline a good local anesthetic choice. The spinal cord is an important site for TCA effects. NE and 5HT neurotransmitters have a substantial role in the inhibition of sensory transmitters at the level of the spinal cord because TCAs inhibit reuptake themselves. Cohen found that intrathecal injection of amitriptyline via inhibition of NMDA could decrease heat hyperalgesia in animal rats (9). In an animal study, Kawamato and colleagues found that intrathecal Desipramine could cause analgesia. This is probably due to one of the other effective mechanisms in proprioceptive, motor, and sensory blocks: sodium channel blockade (10). The analgesic effects of TCAs could be due to inhibition of the NMDA receptor or drug tendencies toward opioid, histamine, and acetylcholine muscarinic receptors. The variation in effects across sites is due to supraspinal, spinal, or peripheral effects of these drugs and a broad range of their reactions after systemic usage (11). Animal studies have shown that amitriptyline is a strong and long-acting local anesthetic when used subcutaneously, intrathecally, or for sciatic nerve block. In Gerner et al.’s (2003) study, other TCAs such as doxepin, imipramine, and trimipramine have had significant effects on sciatic nerve block, which have shown effects similar to those of doxepin in our study (11). Sudah found similar results after amitriptyline injection in the sciatic nerves of rat subjects (7). Other studies observed that secondary amines such as nortriptyline and amoxapine could have anesthetic effects but weaker than tertiary amines (1, 12, 13). In another study, Gerner (2005) examined the anesthetic effects of amitriptyline after skin-patch usage and found that the skin anesthesia effect of this drug was comparable with the effect of lidocaine (14). Consequently, the probability of local anesthetic effects for these drugs has been mentioned frequently in the literature. Also medical practitioners can use doxepin for different procedures such as IV-line access, vaccination, circumcision, skin procedures, skin graft, and neuropathic pain treatment (5). Some studies have found that the impulse-blocking abilities of doxepin are similar to the impulse-blocking abilities of amitriptyline. Doxepin also has shown longer duration blocks on the sciatic nerves of rat model in comparison with bupivacaine. These two findings were not compatible with our trial results. Also, compared with other TCAs, doxepin has longer analgesic effects with fewer cardiac toxicities. Some studies have shown that intrathecal injection of amitriptyline could decrease excitatory amino acids such as aspartate, glutamate, and Interleukin-1 beta and 6 in the cerebrospinal fluid (CSF) of studied rats (15, 16). Just like local anesthetics, the effects of TCAs in these studies appeared in the first hour after intrathecal injection. Considering the results of similar studies, one comes to the understanding that amitriptyline and doxepin are worthy local anesthetics (17). In our study, 3 rats died after intrathecal injection. Their deaths were most likely due to cardiac toxicity and arrest via blocking sodium channels in heart myocytes. In another study, Ogatta found that TCAs’ effects were stronger with amitriptyline (18). At the time of this writing, there is no meta-analysis on the maximum allowable dose of intrathecal injection of this drug group, and no studies have been conducted on humans (all existing studies have been conducted in animal labs). Further investigation is needed for this drug group to evaluate the safety of these drugs for humans. The authors of this article encourage researchers to evaluate different drugs and regimens of TCAs in animal and human studies.
